# Persequi-Nitrate of Iron, in Chronic Diarrhœa

**Published:** 1852-11

**Authors:** Henry A. Ramsay

**Affiliations:** Thompson, Georgia


From Nelson's Northern Lancet.

*Persesqui-Nitrate of Iron, in Chronic Diarrhœa, by* HENRY A. RAMSAY, M. D.

DR. NELSON.

Dear Doctor :—The above preparation has never received the attention from southern physicians, in the management of those chronic affections of the bowels so frequently encountered in this section of the Union, which its virtues entitle it to. The old and unphilosophical practice of administering the *immortal* Blue Pill in all intestinal diseases incident to this climate, still remains much in vogue. Practitioners of enlarged experience, and of fine reputation, continue to entertain the idea that the *Liver* is at fault in all these cases, and that the mercurial treatment is the *magnum remedium* for their relief. For our own part, we are not pent up in such bounds, we harbor not such restricted ideas; and we have not, for some years, believed the Liver amenable for half the morbid crimes alleged against it; and we hope to live to see the period when this Liver infatuation will become modified among southern medical men.

It was, we believe, a German physician who first directed the attention of the profession to the virtues of the Persesqui-nitrate of Iron, in chronic diarrhœal discharges, consequent upon a perverted condition of the mucous membrane of the intestinal canal, which in southern parlance would be construed into a vitiated biliary secretion. We are not so dogmatical or exclusive, as to suppose that the liver is never in error in such affections; but, on the contrary, we believe this organ to be in a morbid state in many forms of diarrhœa here, though not in every case. We see, in southern latitudes, chronic affections of the bowels dependent upon a perverted biliary secretion; upon excessive stimulation of the stomach and its appendages; or produced by erroneous dietetic regulations, by the abuse of the mercurial practice, and by an improper indulgence in fruits; while in other cases, no appreciable causes can be traced. There is generally, in this affection; a tenderness of the bowels; the tongue is red; the pulse quick; tenderness of the epigastrium; a pale, and emaciated appearance; the bowels are loose, and the evacuations liquid. The majority of these cases are of long standing, and have been dosed *usque ad nauseam*, until the sensibility of the nervous distributions to the stomach and intestines have become inert, and almost unsusceptible to the action of remedial agents. It is in such cases that the Persesqui-nitrate of Iron displays its powerful curative properties. But it is by no means inapplicable to cases less sensitive, or of more recent date. We have employed no remedy from which we have derived so good an effect, as a general prescription, in chronic diarrhœal discharges of the South, and we can confidently recommend this preparation of Iron, as a safe and efficient medicine.—In those pale-looking patients, with red tongues, and a constant looseness—which eminently portrays an impoverished condition of the sanguiferous system, it will act like a charm, restoring the wonted tone of the stomach, and retrieving the impaired functions of the liver and intestinal tube.

We remember a case in point that occurred in our practice last Spring, in the person of one of our most worthy and respectable citizens. He had suffered for eighteen months, prior to our seeing him, with chronic diarrhœa; he was anæmic, his frame was rapidly sinking under the effect of numerous daily discharges; his features were contracted; his muscular system wasted; his temper irritable; the tongue red; the pulse quick and feeble; in a word, in point of health he was "doing a sinking business." The infallible Blue Pill had had a fair and faithful trial, and though of no avail, was still continued. Many distinguished men in various parts of the State had been consulted, and the prescription was only reiterated or conjoined with Nitric Acid, or the Nitro-muriatic acid bath.—When we visited this patient he was despondent, but we insisted upon a trial of the remedy, which was acceded to. He was ordered ten drops, three times a day, for ten days, the quantity then to be increased to twelve. The first ten days brought a decided improvement, and a diminution of the evacuations; the second ten only magnified the apparent benefit; and while we are writing, the patient is attending to the agricultural pursuits of the season, which he has not done for eighteen months. He is, indeed, a monument to the power and influence of Persesqui-nitrate of Iron, in chronic diarrhœa, with perverted nervous sensibilities. To be of permanent benefit, the remedy should be persisted in for some time. It is a safe and excellent agent in cases such as we have described; and we trust that other members of the profession will record the results of their experience with it. We prescribe ten drops of the solution of the Persesqui-nitrate of Iron, in a tablespoonful of water. This is given three times a-day to adults, and the quantity gradually increased to fifteen or eighteen drops. If this should happen to meet the eye of any medical reader, having in his care a hopeless case of chronic diarrhœa, let him try this preparation. We do not pretend to say that it will prove a specific in every instance; but that it will act beneficially in a large number of cases that have proved rebellious in other plans of treatment, we entertain not the least doubt. It does not occasion nausea, or any other unpleasant symptoms. Should the liver be perverted, it will exert a healthy influence over it. If the blood is impoverished, it adds to its solid particles, increases the appetite, and improves the digestive function. We administer it independent of cathartics, relying upon it singly, for we could never see the utility of repeatedly purging a patient, to cure him of a disease, the characteristic symptom of which is purging.

Very respectfully and truly yours,

HENRY A. RAMSAY, M. D.

Thompson, Georgia, September, 1852.

